# Endovascular Recanalization and Standard Medical Management for Symptomatic Non-acute Intracranial Artery Occlusion: Study Protocol for a Non-randomized, 24-Month, Multicenter Study

**DOI:** 10.3389/fneur.2021.729534

**Published:** 2021-09-28

**Authors:** Huijun Zhang, Jianjia Han, Xuan Sun, Zhongrong Miao, Xu Guo, Guodong Xu, Yaxuan Sun, Chao Wen, Chaobin Wang, Yingchun Wu, Yaoming Xu, Yuanfei Jiang, Shiyong Zhang, Chao Liu, Di Li, Yan Liu, Chenghua Xu, Feng Gao

**Affiliations:** ^1^Department of Neurology, Tong Ren Hospital Shanghai Jiaotong University School of Medicine, Shanghai, China; ^2^Department of Interventional Neuroradiology, Beijing Tiantan Hospital, Beijing, China; ^3^Department of Neurology, Beijing Anzhen Hospital, Beijing, China; ^4^Department of Neurology, Hebei General Hospital, Shijiazhuang, China; ^5^Department of Neurology, Shanxi Provincial People's Hospital, Taiyuan, China; ^6^Department of Neurology, Taiyuan Central Hospital, Taiyuan, China; ^7^Department of Neurology, Liangxiang Teaching Hospital, Beijing, China; ^8^Department of Neurology, ORDOS Central Hospital, Ordos, China; ^9^Department of Neurology, TongLiao City Hospital, Tongliao, China; ^10^Department of Neurology, Tai'an Hospital of Traditional Chinese Medicine, Tai'an, China; ^11^Department of Interventional Neurology, Beijing You'anmen Hospital, Beijing, China; ^12^Department of Neurology, Handan Central Hospital, Handan, China; ^13^Department of Neurology, Dalian Municipal Central Hospital, Dalian, China; ^14^Department of Neurology, Jingjiang People's Hospital, Taizhou, China; ^15^Department of Neurology, Taizhou First People's Hospital, Taizhou, China

**Keywords:** symptomatic non-acute intracranial artery occlusion, standard medical therapy, endovascular recanalization, major and mild stroke, primary and secondary outcomes

## Abstract

**Background:** The management of patients with symptomatic non-acute intracranial artery occlusion (sNA-ICAO), which is a special subset with high morbidity and a high probability of recurrent serious ischemic events despite standard medical therapy (SMT), has been clinically challenging. A number of small-sample clinical studies have also discussed endovascular recanalization (ER) for sNA-ICAO; however, there is currently a lack of evidence from multicenter, prospective, large-sample cohort trials. The purpose of our present study was to evaluate the technical feasibility and safety of ER for sNA-ICAO.

**Methods:** Our group is currently undertaking a multisite, non-randomized cohort, prospective registry study enrolling consecutive patients presenting with sNA-ICAO at 15 centers in China between January 1, 2020 and December 31, 2022. A cohort of patients who received SMT and a cohort of similar patients who received ER plus SMT were constructed and followed up for 2 years. The primary outcome is any stroke from enrollment to 2 years of follow-up. The secondary outcomes are all-cause mortality, mRS score, NIHSS score and cognitive function from enrollment to 30 days, 3 months, 8 months, 12 months, 18 months, and 2 years of follow-up. Descriptive statistics and linear/logistic multiple regression models will be generated. Clinical relevance will be measured as relative risk reduction, absolute risk reduction and the number needed to treat.

**Discussion:** The management of patients with sNA-ICAO has been clinically challenging. The current protocol aims to evaluate the technical feasibility and safety of ER for sNA-ICAO.

**Trial Registration Number:** www.ClinicalTrials.gov, identifier: NCT04864691.

## Background

Large intracranial artery occlusion is a major cause of stroke and is associated with a high risk of stroke recurrence and poor stroke outcome, especially in China (1, 2). For symptomatic non-acute intracranial artery occlusion (sNA-ICAO) (within 24 h to 6 months), some patients continue to be symptomatic despite standard medical therapy (SMT) (3–5). Extracranial-intracranial (EC-IC) artery bypass surgery fails to show benefits in preventing ischemic attacks or ischemic stroke when performed for sNA-ICAO (6, 7). The optimal treatment for patients with sNA-ICAO disease remains undefined. Currently, SMT, including an antiplatelet regimen and risk factor management, has been used to treat patients with sNA-ICAO disease. Unfortunately, the natural course of this condition shows that these patients often experience recurrent symptoms despite SMT. Recently, a series of small-sample clinical studies have reported that endovascular recanalization (ER) is feasible for sNA-ICAO (8–13). However, most of the previous studies are based on small-sample, single-center retrospective analyses, and there is no high-level evidence from large multicenter samples or prospective studies to indicate the effectiveness and safety of ER for sNA-ICAO.

Therefore, we launched a prospective registry study of patients with sNA-ICAO from 15 centers in China to test whether ER combined with SMT is superior to SMT alone in the primary prevention of stroke in patients with symptomatic non-acute cerebral artery occlusion.

## Methods And Design

### Study Design and Setting

The trial was retrospectively registered on ClinicalTrials.gov on April 25, 2021, with reference number NCT(04864691). The study is a multicenter, prospective registry, non-randomized cohort study sponsored by professor Feng Gao of Beijing Tiantan hospital to assess patients affected by sNA-ICAO undergoing ER and SMT. This protocol was developed according to the Standard Protocol Items: Recommendations for Interventional Trials (SPIRIT) Statement. Fifteen centers across China will participate in the study and provide data. All centers have a similar perioperative pathway and use enhanced recovery after surgery (ERAS) protocols. The participating centers are as follows: Department of Interventional Neuroradiology, Beijing Tiantan Hospital; Department of Neurology, Tong Ren Hospital Shanghai Jiaotong University School of Medicine; Department of Interventional Neurology, Beijing You 'anmen Hospital; Department of Neurology, Beijing Anzhen Hospital; Department of Neurology, Hebei General Hospital; Department of Neurology, Shanxi General Hospital; Department of Neurology, Taiyuan Central Hospital; Department of Neurology, Liangxiang Hospital; Department of Neurology, ORDOS Central Hospital; Department of Neurology, TongLiao City Hospital; Department of Neurology, Tai'an Hospital of Traditional Chinese Medicine; Department of Neurology, Handan Central Hospital; Department of Neurology, Dalian Municipal Central Hospital; Department of Neurology, Jingjiang people's Hospital; Department of Neurology, Taizhou first people's Hospital.

### Participants

**Figure 1:**
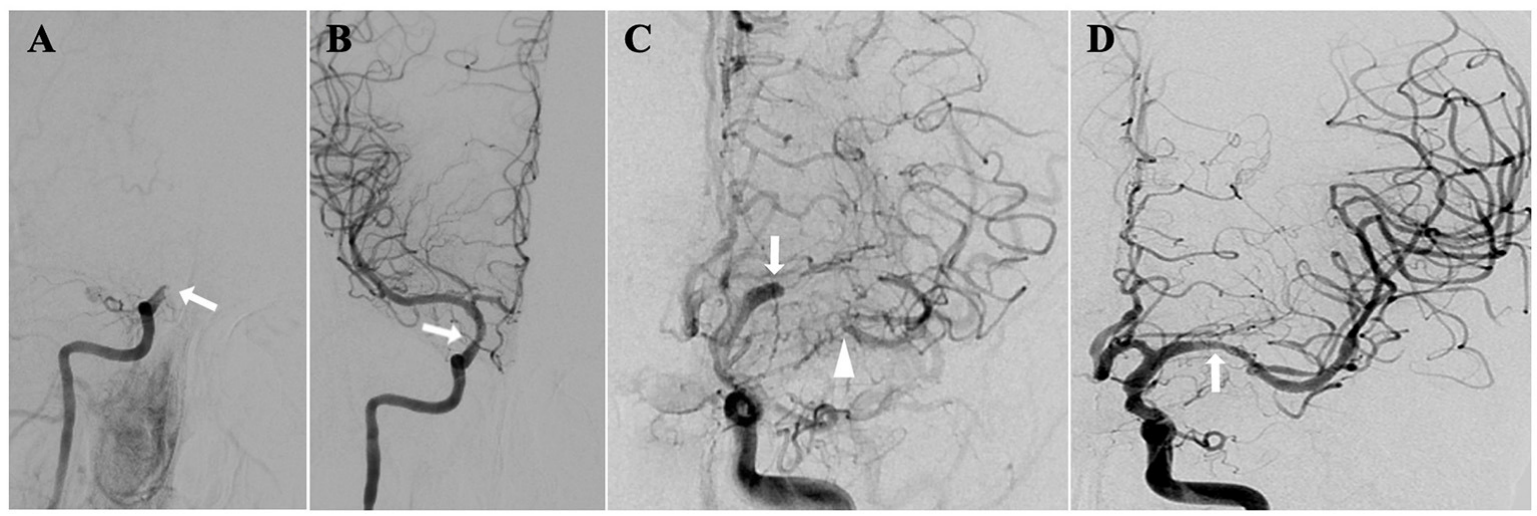
sNA-ICAO diagnosed by DSA. **(A)** Illustrations of non-acute occlusion of intracranial segment of internal carotid artery; **(B)** recanalization after endovascular treatment; **(C)** illustrations of non-acute occlusion of middle cerebral artery; **(D)** recanalization after endovascular treatment.

We will include patients with imaging (MRA/CTA/DSA) and clinical diagnosis of sNA-ICAO (Figure 1) between January 1, 2020 and December 31, 2022 in the participating centers. Eligibility screening will be performed by the principal investigator in accordance with the inclusion/exclusion criteria (Tables 1, 2). Based on the patient's previous history, imaging features of the lesion and the attitudes of the patient and family members, the local investigative team in each center will determine whether to give SMT plus SMZ or SMT alone. Both groups of patients share general inclusion/exclusion criteria and primary and secondary endpoints.

**Table 1:** Primary inclusion criteria for participants in the trial.

1. Patient age ≥ 18 years old.
2. Symptomatic sNA-ICAO defined as:
• Diagnosed by CTA or MRA and confirmed by angiography;
• Vascular occlusion time more than 24 h;
• TIA or ischemic stroke (confirmed by CT or MRI) related to the LCAO despite SMT <90 days prior to enrollment.
3. Modified Rankin scale score of 0 or 1 at the time of informed consent.
4. More than one risk factor for atherosclerosis.
5. For patients with ICA or MCA M1 segment occlusion, ipsilateral hypoperfusion confirmed by CTP or MRI perfusion imaging prior to enrollment and analysis by the RAPID system.
6. For patients with intracranial segment occlusion of the vertebral artery, severe stenosis or occlusion of the contralateral vertebral artery.
7. Among women, no childbearing potential; or if a woman with childbearing potential, a negative pregnancy test result prior to admission.
8. Agreement of the patient to comply with all protocol-specified follow-up appointments.
9. All enrolled patients refused bypass surgery.
10. Signature by a patient of a consent form that has been approved by the local governing institutional review board (IRB)/medical ethics committee (MEC) of the respective clinical site.

**Table 2:** Primary exclusion criteria for participants in the trial.

1. Intolerance or allergic reaction to a study medication without a suitable management alternative.
2. No atherosclerotic intracranial vasculopathies, such as dissection, moyamoya disease and vasculitis.
3. Concomitant intracranial aneurysms or any bleeding disorder.
4. Life expectancy <1 year due to other medical conditions.
5. Large infarction core, defined as an ASPECTS <6 in anterior circulation and pc-ASPECTS <6 points in posterior circulation.
6. For patients with MCA M1 segment occlusion, concomitant ≥50% stenosis of the proximal internal carotid artery or other intracranial arteries.
7. For patients with intracranial segment occlusion of the vertebral artery, continuance of the occluded vertebral artery to the posterior inferior cerebellar artery with no stump.
8. Incomplete clinical and imaging data.
9. Coexistent cardioembolic source (e.g., atrial fibrillation, mitral stenosis, prosthetic valve, MI within six weeks, intracardiac clot, ventricular aneurysm and bacterial endocarditis).
10. Occlusive lesions with severe calcification.
11. Platelet count <100,000/ml or history of heparin-induced thrombocytopenia.
12. Left ventricular ejection fraction <30% or admission for heart failure in the prior 6 months.
13. Extreme morbid obesity that would compromise patient safety during the procedure or the periprocedural period.
14. Coronary artery disease with two or more proximal or major diseased coronary arteries with 70% stenosis that have not or cannot be revascularized.
15. Anticoagulation with Marcumar, warfarin or direct thrombin inhibitors or anti-XA drugs.
16. Chronic atrial fibrillation.
17. Any history of atrial fibrillation or paroxysmal atrial fibrillation in the past 6 months that is considered to require long-term anticoagulant therapy.
18. Other high-risk cardiogenic embolisms, including left ventricular aneurysm, severe cardiomyopathy, aortic or mitral mechanical heart valve, severe calcified aortic stenosis (valve area <1.0 cm2), endocarditis, moderate to severe mitral stenosis, left atrial thrombus or any intracardiac mass or known paradoxical embolism of unrepaired PFO.
19. Unstable angina defined as rest angina with ECG changes that is not amenable to revascularization (patients should undergo planned coronary revascularization at least 30 days before randomization).
20. Any major surgery, major trauma, revascularization procedure or acute coronary syndrome within the past 1 month.
21. serum creatinine > 2.5 mg/dl or estimated GFR <30 cc/min.
22. Major surgery planned within 3 months after enrollment.
23. Currently listed or being evaluated for major organ transplantation (i.e., heart, lung, liver and kidney).
24. Participation in other trials and may affect the results of this study.
25. Inability to understand and cooperate with research procedures or provide informed consent.
26. Endarterectomy, bypass or stent implantation performed on the proximal end of the occlusion vessel.

### Ethical Issues

Data collection will be performed according to the World Medical Association Declaration of Helsinki. All the patients gave written informed consent to participate. Ethical permission was received from Beijing Tiantan Hospital, the Capital Medical University Medical Ethics Committee (number: KY2020-114-02), and the institutional review boards of all partner sites. The standard of care for patients participating in this study will remain the same.

### SMT

Sites implemented SMT for all patients with guidance from the Medical Management Core. Patients in SMT group will take aspirin (100 mg/day) and clopidogrel (75 mg/day) for 90 days followed by lifelong aspirin or clopidogrel monotherapy thereafter. The primary risk factors of cerebrovascular disease including systolic blood pressure and LDL cholesterol, will be controlled in line with protocols. Systolic blood pressure will be controlled below 140 mmHg or 130 mmHg in patients with diabetes and LDL will be controlled below 70 mg/dl with Atorvastatin (14). At each follow-up visit, blood pressure and LDL will be tested, and if the standard is not met, the medication will be adjusted based on the measurements. Management of secondary risk factors such as diabetes, non-HDL cholesterol, smoking, weight and physical activity will be coordinated with the patient's primary physician or other consultant as needed. A lifestyle modification program, INTERVENT, will be provided to each patient.

### ER Protocol

A dual antiplatelet regimen with acetylsalicylic acid (100 mg) and clopidogrel (75 mg) is started at least 3 days before the procedure. All procedures are performed under general anesthesia by an experienced interventional neuroradiologist. After placement of sheath introducers, heparin is given intravenously to maintain the coagulation time between 200 and 300 s. The 6- or 8-French guiding catheter is located distal to the occluded artery as much as possible. Under the route map, the micro guidewire in combination with a microcatheter and the microcatheter are used to carefully pass through the occluded segment. Angiography with the microcatheter should confirm that the guidewire is in the true lumen. The exchange micro guidewire is then sent into the micro catheter, and the microcatheter is exchanged out. The balloon catheter is advanced smoothly into the occluded segment along the exchange micro guidewire. After the occluded segment is dilated with the balloon, angiography with a guiding catheter is performed. Stents are deployed in cases of residual severe stenosis, vascular dissection and failure to maintain forward flow (according to the judgment of the neuroradiologist to select the stent). If one stent cannot completely cover the lesion, multiple stents can be implanted. Successful revascularization is defined as a modified TICI grade 2b or 3 and residual stenosis <50%.

For patients with ICA or MCA M1 segment occlusion, ipsilateral hypoperfusion should be confirmed by CTP or MRI perfusion imaging prior to enrollment according to a previous study (15). Moreover, the non-contrast and perfusion scans are additionally transferred to the Rapid Processing of Perfusion and Diffusion (RAPID) system, providing analysis of perfusion source images with respect to the DEFUSE 3 criteria (16).

Periprocedural drug therapy is shown in Table 3. After the procedure, if there are no hemorrhagic complications on the head CT scan, intravenous anticoagulation or antiplatelet therapy is continued for at least 24-48 h. Then, dual antiplatelet therapy is maintained for 3-6 months followed by lifelong aspirin or clopidogrel monotherapy thereafter.

**Table 3:** Periprocedural drug therapy.

**Medication**	**Preprocedure**	**Intraprocedure**	**Postprocedure**	**Postdischarge**
Heparin	None	Maintain ACT 250–300 s	None	None
Aspirin	300 mg p.o. q.d. (Begin 72 h before)	None	100 mg p.o. q.d. (Begin 24 h later)	100 mg p.o. q.d. for 360 days
Clopidogrel	300 mg p.o. q.d. (Begin 72 h before)	None	75 mg p.o. q.d. (Begin 24 h later)	75 mg p.o. q.d. for 360 days
Cilostazol (Clopidogrel resistance)	100 mg p.o. b.i.d. (begin 72 h before)	None	100 mg p.o. b.i.d. (Begin 24 h later)	100 mg p.o. b.i.d. for 360 days
Tirofiban	None	PRN	0.15 μg/kg/min for 24 h	None
Atorvastatin (or dose equivalent of another statin)	Total of 40/80 mg	None	40/80 mg p.o. q.d	40/80 mg p.o. q.d

### Data Design and Management

Data design and management is the responsibility of the Scientific Committee of Capital Medical University experts. They will keep watch on the database and propose amendments at any time to achieve the purpose of the study. We will collect patient information in a confidential manner in line with China privacy laws. Each center will be in charge of the personal data collected related to the study. Then each patient will be assigned an anonymous identification code. In each center, a responsible physician will registered the information of every enrolled patients on the internet-based data storage file. Each center has its own account and password, and each center can only see patient information uploaded by their own center when they access the web database; if a center research investigator wants to see information on all patients enrolled in their study, they need to request it from the Scientific Committee. All the data will be analyzed anonymously by a statistician.

**Figure 2:**
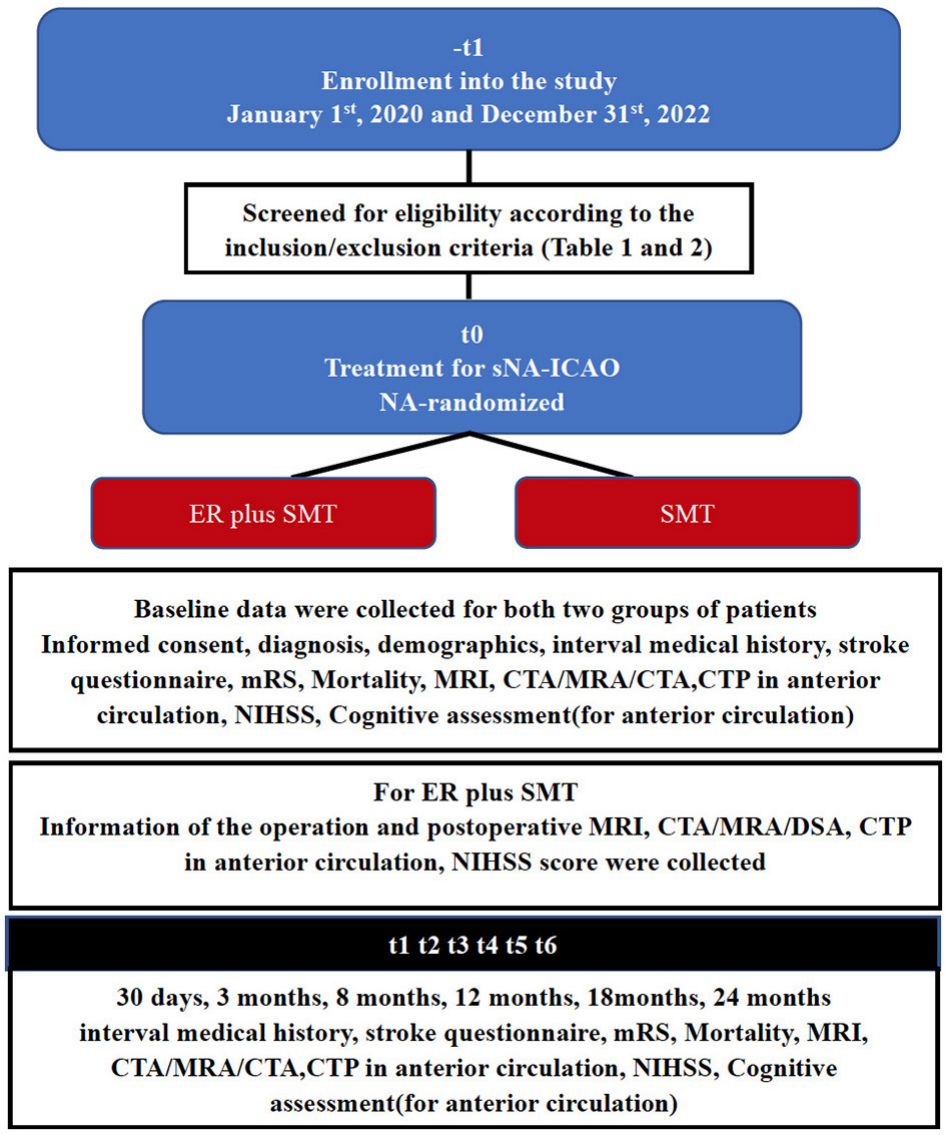
Flowchart of the trial design.

Information of baseline including demographics, vascular risk factors (such as diabetes mellitus, arterial blood pressure, hyperlipidemia, cardiac disease, and smoking) and stroke symptoms [with the Questionnaire for Verifying Stroke-free Status (QVSS) (17), the modified Rankin Scale (mRS) (18) and the National Institutes of Health Stroke Scale (NIHSS) (19)], including morphology occlusion stump, occlusion to recanalization, last symptom to recanalization and cognitive testing (in anterior circulation) were collected (Figure 2).

For patients with ICA or MCA M1 segment occlusion, Cognitive function assessment related to vascular cognitive status, is performed at baseline, 30 days, 3 months, 8 months, 12 months, 18 months, and 24 months. The assessment consists of five tests covering the following four domains of cognitive function: the word list learning test and the delayed recall test from the Chinese version of the Alzheimer's Disease Assessment Scale-cognitive subscale (ADAS-Cog) (20), executive function/processing speed (animal naming and letter fluency), and attention/working memory (digit span) (Figure 2).

Figure 2 illustrates the follow-up schedule. If the patient is unable to come to the hospital for a face-to-face follow-up visit, a telephone follow-up visit will be conducted if possible and will include a brief medication history, any ischemic events, daily functioning and changes in cognitive function.

### Primary and Secondary Outcomes

In this trial, the primary outcome is any stroke from enrollment to 2 years of follow-up. The secondary outcomes are all-cause mortality, mRS score, NIHSS score and cognitive function from enrollment to 30 days, 3 months, 8 months, 12 months, 18months, and 2 years of follow-up. Stroke will be defined as rapidly developing clinical signs of focal disturbance of cerebral function lasting more than 24 h with no apparent cause other than that of vascular origin according to the World Health Organization (21). Outcome will be determined by an adjudication committee that is unaware of the trial design and grouping.

Major stroke is defined as the NIHSS score is ≥6 at least 30 days after the date of stroke onset, mild stroke is defined as deterioration in the NIHSS score is ≤ 4 points and bright spots appear on the brain DWI or as determined by the Stroke Adjudication Committee according to clinical data.

### Statistical Analysis

Based on data from previous studies (6, 9), the 2-year incidence rates of ipsilateral ischemic stroke in ER plus SMT and SMT alone are approximately 10 and 20%, respectively. Assuming a two-sided significance level of 5%, a power of 80%, non-participating rate of 20% and dropout rate of 20%, the requirement for the ER plus SMT and SMT alone group was calculated to be 160 and 320 patients, respectively (1:2 allocation). Reviewing previous studies (8–13), we took the preoperative complication (arterial dissection, arterial perforation, thrombus translocation, subacute stent thrombosis, hemorrhage, and died) rate >15% as the termination criteria.

Prior to statistical analysis, the statistician will collate the data. If he finds any missing data, he will contact the responsible doctor and ask him to check the medical records of the patient visits and follow-up visits to clarify whether the missing information is in the data sheet. If the missing data cannot be obtained, we will conduct multiple imputation under a multivariate normal distribution to impute missing outcome data in the primary analysis of all outcomes, with a sensitivity analysis on complete cases only. Continuous variables are expressed as medians and interquartile ranges (IQRs) and as absolute numbers and percentages, while categorical variables are expressed as the means and standard deviations (SDs). Shapiro–Wilk test, histogram, and QQ chart were used to confirm normal distribution of data. We use chi-squared test, *t*-tests and Mann–Whitney *U*-test to compare categorical variables, continuous variables and scores, respectively. Using cox proportional regression models with 95% confidence intervals to test the risk of mortality or Ischemic events. Adjusted estimates of outcome (common odds ratio, odds ratio, and β) will be calculated by taking the following variables into account: age, baseline NIHSS and mRS score, baseline cognitive function, sex, medical history, ischemic stroke, duration from last neurologic event and occlusion site. For propensity score matching analysis, we will perform 1:1 matching based on the nearest-neighbor matching algorithm with a caliper width of 0.2 of the propensity score with age, baseline NIHSS and mRS score, baseline cognitive function, location of occlusion and medical history questionnaire. All statistical analyses will be performed with SPASS 25.0, and *P* < 0.05 will be considered significant.

### The Responsibilities of the Scientific and Steering Committees

The responsibilities of the Scientific Committee is to supervise the publication and presentation of the final research results on the academic symposium, in consultation with the Steering Committee. The Committee will make sure that all publications adhere to authorship guidelines. Members are Xuan Sun, and Miao Zhongrong.

The Steering Committee will be responsible for the planning and implementation of the registry. Specifically, they: approve the participating centers and the corresponding doctors in each center; perform quality control of the data; direct and propose amendments at any time to achieve the purpose of the study; analyze and revise the final results for submission to the congress and publication in a scientific papers. Members are Feng Gao; Xu Guo; Chao Wen; Hui-Jun Zhang.

## Discussion

### Summary

The management of patients with sNA-ICAO, which is a unique subset with high morbidity and a high probability of recurrent serious ischemic events despite maximal medical therapy, has been clinically challenging. Some small-sample clinical studies have also discussed endovascular recanalization for sNA-ICAO; however, there is no evidence from multicenter large-sample trials. The aim of our present study is to evaluate the technical feasibility and safety of non-acute intracranial artery occlusion. We will perform subgroup analysis according to the angiographic classification of sNA-ICAO proposed by our previous studies (9, 13), stump morphology, duration from occlusion confirmed by imaging and clot characteristics evaluated by High Resolution MRI (optional examine).

### Limitations

Non-randomized of the treatment arms is the main limitation of the present research. Clinical reasoning behind treatment choice may affect conclusions, but the extensive data collection of numerous potentially relevant factors will allow us to adjust for potential confounders. Comparative effectiveness trials such as this are valuable because both physicians and patients have a complex range of factors and decisions that affect their treatment options, which cannot be assessed in RCTs. Another limitation of the study design is that our present study population is limited to the Chinese patients which restrains generalizability of the results to other populations/ethnicities. For this limitation, we will perform our further studies which will include patients from other countries.

### Strengths and Relevance

Our present study have several advantages except for non-randomization. Current studies on endovascular treatment of ICAS are mainly from a number of single-center, small-sample, single-arm retrospective analyses. First, our study is prospectively designed and will contain the largest sample size to date. Second, our study is a multicenter design across 15 provinces of the country, thus minimizing selection bias; finally, we will also compare the results of ER combined with SMT and SMT alone for sNA-ICAO. Importantly, we planned a long-term follow-up of 24 months, which will allow us to examine both short-term and long-term outcomes of patients.

## Ethics Statement

We will follow the World Medical Association Declaration of Helsinki to collect data and all potential participants will be required to sign an informed consent form. Ethical permission was received from the Institutional Review Board of Beijing Tiantan Hospital (number: KY2020-114-02).

## Author Contributions

FG, XG, CWe, and HZ are on the Scientific Committee for the current project. XS and ZM are on the steering committee for the current project. HZ, XS, FG, XG, GX, YS, CWe, CWa, YW, YX, YJ, SZ, CL, DL, YL, and CX will provide patient information for the multicenter trial. HZ, XS, and FG wrote the draft. All authors were involved in the design of the protocol, revised the draft, and approved the final manuscript.

## Funding

This study was funded by National Key R&D Program (2018AAA0102600).

## Conflict of Interest

The authors declare that the research was conducted in the absence of any commercial or financial relationships that could be construed as a potential conflict of interest.

## Publisher's Note

All claims expressed in this article are solely those of the authors and do not necessarily represent those of their affiliated organizations, or those of the publisher, the editors and the reviewers. Any product that may be evaluated in this article, or claim that may be made by its manufacturer, is not guaranteed or endorsed by the publisher.

## Abbreviations

sNA-ICAO: symptomatic non-acute intracranial artery occlusion
SMT: standard medical therapy
ER: endovascular recanalization
EC-IC: Extracranial-intracranial
SPIRIT: Recommendations for Interventional Trials
ERAS: enhanced recovery after surgery
IRB: institutional review board
MEC: medical ethics committee
RAPID: Rapid Processing of Perfusion and Diffusion
ADAS-Cog: Alzheimer's Disease Assessment Scale-cognitive subscale.
